# Protocol for studying topological DNA interactions by purified fission yeast condensin

**DOI:** 10.1016/j.xpro.2024.102995

**Published:** 2024-04-04

**Authors:** Minzhe Tang, Frank Uhlmann

**Affiliations:** 1 Chromosome Segregation Laboratory, The Francis Crick Institute, London NW1 1AT, UK

**Keywords:** Model Organisms, Molecular Biology, Protein Biochemistry, Protein expression and purification

## Abstract

To understand the transition from interphase chromatin into well-shaped chromosomes during cell divisions, we need to understand the biochemical activities of the contributing proteins. Here, we present a protocol to investigate how the ring-shaped condensin complex sequentially and topologically entraps two DNA substrates. We describe the steps to prepare purified *Schizosaccharomyces pombe* condensin, as well as bulk biochemical assays to monitor the first and second DNA capture reactions. This protocol may facilitate further investigations of these essential genome organizers.

For complete details on the use and execution of this protocol, please refer to Tang et al.^1^

## Highlights

• Biochemically probing topological protein-DNA interactions by *S. pombe* condensin
• Rapid screening for optimized subunit co-overexpression in *S. cerevisiae*
• Purification steps to minimize contamination and denaturation
• Adaptable to study other DNA-entrapping proteins

## Abstract (editor highlights)

Publisher’s note: Undertaking any experimental protocol requires adherence to local institutional guidelines for laboratory safety and ethics.

## Graphical abstract

**Figure:**
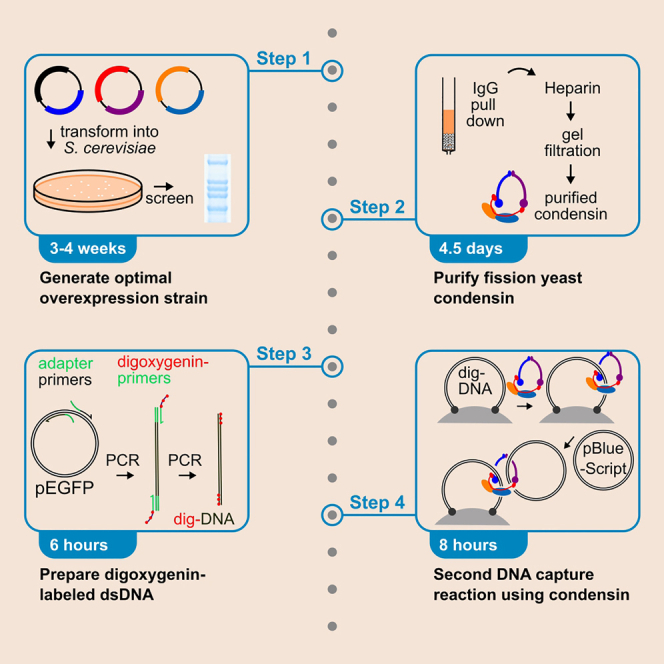

## Before you begin

The protocol below describes the steps to investigate how the chromosomal condensin complex sequentially topologically entraps two double stranded DNA (dsDNA) substrates to establish dsDNA-dsDNA interactions. This protocol can be adapted to study related Structural Maintenance of Chromosome (SMC) complexes such as cohesin, the SMC5-SMC6 complex, or other proteins that might entrap more than one DNA substrate.

Before you begin, note that this protocol requires the use of pBlueScript supercoiled plasmid dsDNA as a DNA substrate. While the plasmid can be purified by various methods, we recommend using CsCl gradient centrifugation^2^ as the DNA purification method for best results. Other purification methods, such as MiniPrep, will generate dsDNA plasmids with a mixture of supercoiled, nicked, and linear topologies. These plasmid preparations will become troublesome especially when you wish to study the impact of DNA topologies in the loading assays described later.

This protocol uses the budding yeast *Saccharomyces cerevisiae* as host cells to ectopically overexpress the fission yeast *Schizosaccharomyces pombe* condensin complex for purification. We observe that, of the five subunits that make up condensin,^3^ the Cnd1 subunit is often present at somewhat substoichiometric levels when compared to the other four subunits in the purified condensin sample. As Cnd1 plays a key role in condensin’s topological loading reaction onto DNA, we recommend selecting for a balanced subunit expression to generate condensin complexes with high specific activity and low non-specific DNA binding. While optimized wild type condensin expression strains have been described,^1^ we describe the method to select such strains in the following to guide researchers to generate any variant condensin complexes in their own studies.

### Construction of a budding yeast strain overexpressing Pk-epitope-tagged fission yeast condensin

**Timing: 3–4 weeks**

1. Clone the cDNA sequences of fission yeast condensin subunits into three integrative budding yeast plasmids under the control of the bidirectional *GAL1-10* promoter.

**Figure 1:**
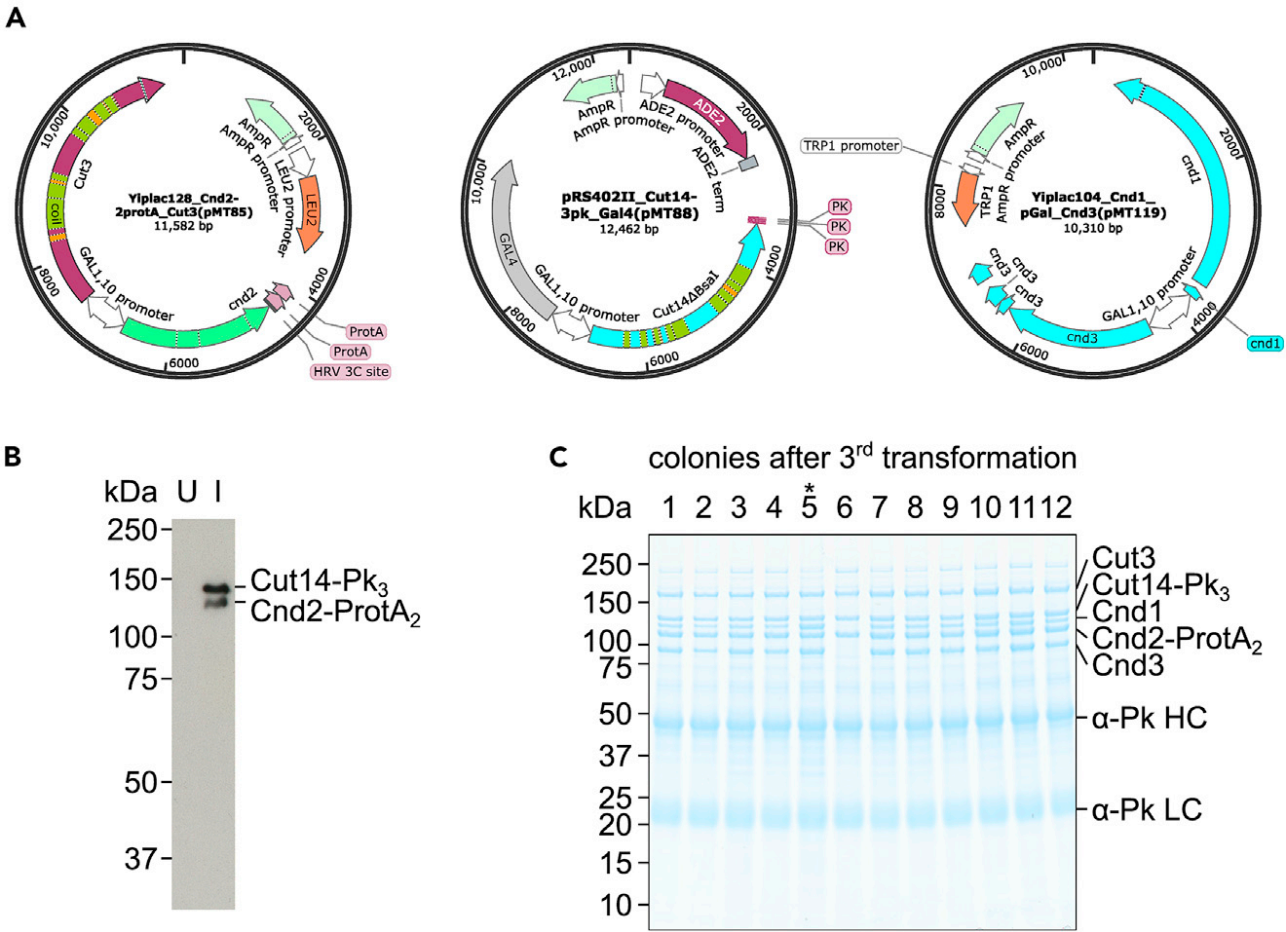
Construction of a budding yeast strain that overexpresses fission yeast condensin (A) Schematic of the three integrative plasmids used to transform budding yeast. Note that fission yeast Cnd3 contains introns, only the cDNA sequences are cloned into the plasmid. (B) Western blot to check protein expression after galactose induction. U, uninduced. I, induced. (C) SDS-PAGE analysis followed by Coomassie Blue staining of the bead-bound protein after small-scale IgG pull down of condensin from different colonies after the third transformation. α-Pk HC and LC, α-Pk tag antibody heavy and light chain, respectively. The asterisk highlights colony 5, which yields a stoichiometric subunit composition of condensin following IgG pull down.

***Note:*** Each plasmid contains one or two of the fission yeast condensin subunits (Figure 1A).

2. Transform to integrate the three plasmids using the standard Lithium acetate protocol,^4^ one at a time, into the genome of the W303 budding yeast strain lacking the abundant vacuolar protease (*pep4Δ*).
  ***Note:****pep4Δ* strain can reduce unwanted protein degradation during cell lysis.
  ***Note:*** We transform the plasmids in the order specified below to facilitate tracking of protein expression during strain construction.
  a. First transformation: pRS402II-*CUT14-Pk*_*3*_*-pGAL1-10-GAL4* at the *ADE2* locus.
    ***Note:*** The Gal4 transcription factor is also expressed under galactose-inducible control, which aids expression of the multiple galactose-controlled condensin subunits.
  b. Second transformation: YIplac128-*CND2-[3C]-ProtA*_*2*_*-pGAL1-10-CUT3* at the *LEU2* locus.
  c. Third transformation: YIplac104-*CND1-pGAL1-10-CND3* at the *TRP1* locus.
3. After the first and second transformation, confirm the galactose-induced over-expression of respective condensin subunits using Western blot from a small-scale culture.
  a. Grow the budding yeast in 20 mL YPR (Yeast Peptone medium supplemented with 2% raffinose) at 25°C overnight (16 h).
  b. On the next day, take 1 mL from the culture, dilute if necessary, and check the OD_600_.
    ***Note:*** We recommend that the culture OD_600_ should not exceed 1.5.
  c. Dilute the culture to OD_600_ = 0.3 using fresh YPR and continue shaking at 25°C for 2 h.
  d. After the 2-h growth, note down an OD_600_ reading and take 2 mL sample from the culture into a 2 mL screw cap cell breaker tube. Process sample according to the protocol below.
    i. Centrifuge at 6,000 × *g* for 1 min at room temperature (25°C).
    ii. Discard supernatant.
    iii. Resuspend cell pellet in 0.5 mL ice-cold 20% TCA (trichloroacetic acid) solution.
    iv. Incubate on ice for at least 10 min.
  e. Induce expression by addition of 2 mL filtered 20% galactose solution into the remaining culture.
  f. Continue shaking at 25°C for 4 h.
  g. After the 4-h induction, note down another OD_600_ reading and take 2 mL sample from the culture. Process this sample as described in step 3d to fix cells in 20% TCA solution and incubate on ice for at least 10 min.
  h. Centrifuge the cell samples in 20% TCA, from before and after galactose induction, at 8,000 × *g* for 1 min. Discard supernatant.
  i. Resuspend pellet in 0.5 mL 1 M Tris-HCl pH 8 solution.
  j. Centrifuge again at 8,000 × *g* for 1 min. Discard supernatant.
  k. Resuspend the cell pellet in 30 μL SDS-PAGE sample buffer.
  l. Boil at 95°C for 5 min.
  m. Add 50 μL acid-washed glass beads.
  n. Rupture cells by vigorous shaking in a bead beater, e.g., MP Biomedicals FastPrep-24, using the default program for *Saccharomyces cerevisiae* cell lysis, i.e., 2 m/s speed for 40 s.
    ***Note:*** Confirm efficient cell breakage by microscopy, adjust breakage cycles if required.
  o. Puncture sample tube and collect lysate into a clean 1.5 mL Eppendorf tube by brief centrifugation as described.^5^
  p. To adjust for the increased cell density following induction, dilute the induced sample 2-fold using SDS-PAGE loading buffer.
  q. Load 8 μL of the samples onto 10% Tris-Glycine SDS-PAGE for separation.
  r. Transfer the protein from SDS-PAGE gel onto a nitrocellulose membrane.
  s. Detect Cut14-Pk_3_ expression using an α-Pk tag antibody (Figure 1B).
    ***Note:*** Cnd2-3C-ProtA_2_ will also be detected by virtue of its protein A tag.

### Screening for stoichiometric condensin subunit overexpression using small-scale purification

**Timing: 1 day**

4. After obtaining colonies from the third transformation, streak at least 10 colonies onto a new selection plate to increase chances of finding a suitable expression strain.
5. Induce condensin expression from each colony in a small-scale culture.
  a. Grow up each colony in 50 mL YPR medium at 25°C until OD_600_ reaches between 0.6 and 1.
  b. Induce protein expression by addition of 5 mL filtered 20% galactose solution.
  c. Continue shaking the culture at 25°C for 4 h.
  d. Centrifuge the culture at 4,000 × *g* at 4°C for 3 min. Discard supernatant.
6. Lyse cells by bead beating.
  a. Resuspend cell pellet in 1 mL ice-cold ddH_2_O and transfer to a 2 mL screw cap cell breaker tube.
  b. Centrifuge the culture at 6,000 × *g* at 4°C for 1 min. Discard supernatant.
  c. Resuspend cell pellet in 0.5 mL ice-cold IgG lysis buffer.
  d. Add 0.5 mL glass beads and lyse cells in a bead beater, e.g., MP Biomedicals FastPrep-24, using the default program for *Saccharomyces cerevisiae* cell lysis. Cool down at 4°C for 5 min.
  e. Repeat the step above two more times.
    ***Note:*** Confirm efficient cell breakage by microscopy, adjust breakage cycles if required.
  f. Puncture sample tube and collect lysate into a clean 2 mL sample tube by brief centrifugation as described.^5^
7. Adsorb condensin to IgG beads.
  a. Add 1 mL ice-cold IgG lysis buffer and mix well with the cell lysate.
  b. Centrifuge at 17,000 × *g* at 4°C for 30 min.
    **CRITICAL:** Less centrifugation time could compromise condensin recovery.
  c. Carefully transfer the clear supernatant phase into a clean low-binding microcentrifuge tube, e.g., a Costar tube. Avoid the white cloudy top layer and the pellet.
  d. Add 10 μL Rabbit IgG agarose beads (20 μL beads suspension) to each sample and rotate on a wheel at 4°C for 1 h.
8. Wash IgG beads with IgG wash buffer.
  a. Centrifuge sample tube at 2000 × *g* at 4°C for 1 min. Discard supernatant.
  b. Resuspend beads in 1 mL IgG wash buffer by inverting tubes. Centrifuge at 2000 × *g* at 4°C for 1 min. Discard supernatant.
  c. Repeat the washing steps above two further times.
9. Analyze condensin-enriched samples using SDS-PAGE.
  a. Resuspend beads in 20 μL SDS-PAGE sample buffer.
  b. Boil at 95°C for 5 min.
  c. Separate 5 μL sample on a 4%–12% gradient Tris-Glycine SDS-PAGE gel.
  d. Coomassie staining.
10. Fission yeast condensin subunits separate well on a 4%–12% gradient gel (Figure 1C). Choose a colony that yields a stoichiometric condensin subunit composition, as indicated by five equal intensity bands following Coomassie Blue staining.
11. Transfer the chosen expression strain as a 15% glycerol stock to −70°C for long term storage.

## Key resources table

**Table:** 

REAGENT or RESOURCE	SOURCE	IDENTIFIER
**Antibodies**		

α-Pk antibody (unless stated otherwise, dilute 1:10,000 for western blot)	Bio-Rad	Cat#MCA1360
α-digoxygenin antibody (not used for western, see main text for dilutions or amount to use)	Abcam	Cat#AB420

**Bacterial and virus strains**		

C2987 chemically competent cells	NEB	Cat#C2987U

**Chemicals, peptides, and recombinant proteins**		

20% trichloroacetic acid (TCA)	Fisher Scientific	Cat#SA254-500
Yeast peptone (YP)	Merck	Cat#68707-500G
Glucose	Merck	Cat#G8270-5KG
Raffinose	Merck	Cat#R0250-500G
Galactose	Merck	Cat#G0625-1KG
Glass beads	Merck	Cat#G8772-500G
Phenylmethylsulfonyl fluoride (PMSF)	Merck	Cat#11359061001
RNase A	Merck	Cat#10109169001
CloneAmp HiFi PCR mix	Takara Bio	Cat#639298
Protein G Dynabeads	Thermo Fisher Scientific	Cat#10004D
Bovine serum albumin (BSA)	Merck	Cat#A4503-100G
LB broth (Millier) (LB)	Merck	Cat#L3522
Tris(2-carboxyethyl)phosphine hydrochloride (TCEP)	Merck	Cat#C4706-10G
IGEPAL CA-630	Merck	Cat#I8896-50ML
Proteinase K	Thermo Fisher Scientific	Cat#25530049
GelRed nucleic acid gel stain	Biotium	Cat#41003-1
StuI	NEB	Cat#R0187S
ScaI-HF	NEB	Cat#R3122S

**Experimental models: Organisms/strains**		

*S. cerevisiae* W303 background *can1-100*, *ura3*, *GAL*, *psi+*, Δ*pep4::HIS3*, *CND2-ProtA*_*2*_-*pGAL1-10*-*CUT3::LEU2*, *GAL4-pGAL1-10-CUT14-Pk*_*3*_*::ADE2*, *CND1-pGAL1-10-CND3::TRP1*	This study	N/A
*S. cerevisiae* W303 background *ade2-1, trp1-1, can1-100, leu2-3,112, ura3, GAL, psi+* Δ*pep4::HIS3*	This study	N/A

**Oligonucleotides**		

MT462: 5′-ggaagcataaagtgtaaagcctgggg CAAATATGTATCCGCTCATGAGACAATAACC-3′	This study	N/A
MT463: 5′- gcttccggctcgtatgttgtgtggaa CCCTTTAGGGTTCCGATTTAGTGC-3′	This study	N/A
MT392: 5’- /5DigN/GCTAGGCATC/iDigN/GCTAGGCATC/iDigN/GGCTCGTATGTTGTGTGG-3′	This study	N/A
MT393: 5’- /5DigN/GCTAGGCATC/iDigN/GCTAGGCATC/iDigN/GCATAAAGTGTAAAGCCTGG-3′	This study	N/A

**Recombinant DNA**		

pEGFP-C1 dsDNA plasmid	Clontech	(discontinued)
pBlueScript dsDNA plasmid	Agilent Technologies	Cat#212205
pRS402II-*CUT14-Pk*_*3*_*-pGAL1-10-GAL4* dsDNA plasmid	This study	N/A
YIplac128-*CND2-[3C]-ProtA*_*2*_*-pGAL1-10-CUT3* dsDNA plasmid	This study	N/A
YIplac104-*CND1-pGAL1-10-CND3* dsDNA plasmid	This study	N/A

**Other**		

FastPrep-24 cell lysis system	MP Biomedicals	Cat#116004500
Screw cap cell breaker tubes	Fisher Scientific	Cat#11452350
Corning Costar low binding plastic microcentrifuge tubes	Fisher Scientific	Cat#10574391
Novex Tris-Glycine mini protein gels, 4%–12%	Thermo Fisher Scientific	Cat#XP04125BOX
Rabbit IgG-agarose bead	Merck	Cat#A2909
Econo-Pac column	Bio-Rad	Cat#7321010
HiTrap heparin HP column	Cytiva	Cat#17040601
Vivaspin 6 concentrator 100,000 MWCO	Cytiva	Cat#28932319
Superose 6 Increase 10/300 GL column	Cytiva	Cat#29091596
NucleoSpin gel and PCR cleanup Kit	Macherey-Nagel	Cat#740609.250
DynaMag-2 magnet	Thermo Fisher Scientific	Cat#12321D
Amersham nitrocellulose western blotting membrane	Cytiva	Cat#10600002

## Materials and equipment

**Table:** IgG wash buffer

Reagent	Final concentration	Amount
Tris-HCl pH 7.5 (1 M)	40 mM	10 mL
NaCl (5 M)	300 mM	15 mL
Glycerol (100% v/v)	10% (v/v)	25 mL
DTT (powder)	2 mM	77 mg
ddH_2_O	N/A	200 mL
**Total**	**N/A**	**250 mL**

Prepare fresh from the stock solutions of each component on the day of the experiment. Keep at 4°C or on ice.

**Table:** IgG lysis buffer

Reagent	Final concentration	Amount
cOmplete protease inhibitor, EDTA free	N/A	1 tablet
PMSF (0.5 M)	0.5 mM	50 μL
RNase A (10 mg/mL)	10 μg/mL	50 μL
IgG wash buffer	N/A	50 mL
**Total**	**N/A**	**50 mL**

Prepare fresh from the stock solutions of each component on the day of the experiment. Keep at 4°C or on ice.

**Table:** SDS-PAGE sample buffer (2x)

Reagent	Final concentration	Amount
Tris-HCl pH 6.8 (1 M)	80 mM	4 mL
SDS (20% w/v)	2% (w/v)	5 mL
Glycerol (100% v/v)	10% (v/v)	5 mL
Bromophenol blue	0.00006% (w/v)	trace
β-mercaptoethanol (omitted for long term storage)	1% (v/v)	0.5 mL
ddH_2_O	N/A	36 mL
**Total**	**N/A**	**50 mL**

Omit β-mercaptoethanol for long term storage. Store at room temperature (25°C) indefinitely. To use, mix 2 mL 2x SDS sample buffer with 20 μL β-mercaptoethanol. Use within 2 months.

**Table:** Heparin A buffer

Reagent	Final concentration	Amount
Tris-HCl pH 7.5 (1 M)	20 mM	10 mL
NaCl (5 M)	100 mM	10 mL
Glycerol (100% v/v)	10% (v/v)	50 mL
DTT (powder)	2 mM	150 mg
ddH_2_O	N/A	430 mL
**Total**	**N/A**	**500 mL**

Prepare fresh from the stock solutions of each component on the day before purification. Degas by applying vacuum after passing through a 0.22 μm filter. Keep at 4°C or on ice.

**Table:** Heparin B buffer

Reagent	Final concentration	Amount
Tris-HCl pH 7.5 (1 M)	20 mM	2 mL
NaCl (5 M)	1 M	20 mL
Glycerol (100% v/v)	10% (v/v)	10 mL
DTT (powder)	2 mM	30 mg
ddH_2_O	N/A	68 mL
**Total**	**N/A**	**100 mL**

Prepare fresh from the stock solutions of each component on the day before purification. Degas by applying vacuum after passing through a 0.22 μm filter. Keep at 4°C or on ice.

**Table:** Gel filtration buffer

Reagent	Final concentration	Amount
Tris-HCl pH 7.5 (1 M)	20 mM	5 mL
NaCl (5 M)	0.2 M	10 mL
Glycerol (100% v/v)	10%	25 mL
DTT (powder)	2 mM	77 mg
ddH_2_O	N/A	210 mL
**Total**	**N/A**	**250 mL**

Prepare fresh from the stock solutions of each component on the day before purification. Degas by applying vacuum after passing through a 0.22 μm filter. Keep at 4°C or on ice.

**Table:** PBS

Reagent	Final concentration	Amount
NaCl	137 mM	8 *g*
KCl	2.7 mM	0.2 *g*
Na_2_HPO_4_	10 mM	1.44 *g*
KH_2_PO_4_	1.8 mM	0.24 *g*
ddH_2_O	N/A	0.95 L
**Total**	**N/A**	**1 L**

Autoclave and store at room temperature (25°C) for a few months.

• PBS-BSA buffer: dissolve 0.5 *g* BSA in 100 mL PBS. Filter through a 0.22 μm filter. Store at 4°C for one month.

**Table:** DNA binding buffer

Reagent	Final concentration	Amount
Tris-HCl pH 7.5 (1 M)	40 mM	8 μL
NaCl (5 M)	50 mM	2 μL
Glycerol (30% v/v)	10% (v/v)	67 μL
TCEP (0.5 M)	1 mM	0.4 μL
EDTA pH 8.0 (0.5 M)	1 mM	0.4 μL
BSA (20 mg/mL)	0.5 mg/mL	5 μL
ddH_2_O	N/A	117.2 μL
**Total**	**N/A**	**200 μL**

Store at −20°C for 3 months.

**Table:** Loading wash buffer

Reagent	Final concentration	Amount
Tris-HCl pH 7.5 (1 M)	40 mM	4 mL
NaCl (5 M)	500 mM	10 mL
Glycerol (100% v/v)	10% (v/v)	10 mL
TCEP (0.5 M)	0.5 mM	0.1 mL
IGEPAL (10% v/v)	0.01% (v/v)	0.1 mL
ddH_2_O	N/A	75.8 mL
**Total**	**N/A**	**100 mL**

Store at 4°C for 2 months.

**Table:** Loading pre-equilibration buffer

Reagent	Final concentration	Amount
Tris-HCl pH 7.5 (1 M)	40 mM	4 mL
NaCl (5 M)	100 mM	2 mL
Glycerol (100% v/v)	10% (v/v)	10 mL
MgCl_2_ (1 M)	5 mM	0.5 mL
TCEP (0.5 M)	0.5 mM	0.1 mL
IGEPAL (10% v/v)	0.01% (v/v)	0.1 mL
ddH_2_O	N/A	83.3 mL
**Total**	**N/A**	**100 mL**

Store at 4°C for 2 months.

**Table:** Loading reaction buffer

Reagent	Final concentration	Amount
Tris-HCl pH 7.5 (1 M)	40 mM	10 μL
KCl (1 M)	40 mM	10 μL
Glycerol (30% v/v)	10%	84 μL
TCEP (0.5 M)	1 mM	0.5 μL
MgCl_2_ (1 M)	3 mM	0.75 μL
BSA (20 mg/mL)	0.1 mg/mL	1.25 μL
ddH_2_O	N/A	143.5 μL
**Total**	**N/A**	**250 μL**

Prepare fresh from the stock solution of each component on the day and keep on ice.

**Table:** DNA elution stock buffer

Reagent	Final concentration	Amount
Tris-HCl pH 7.5 (1 M)	40 mM	4 mL
NaCl (5 M)	50 mM	1 mL
Glycerol (100% v/v)	10%	10 mL
EDTA pH 8.0 (0.5 M)	20 mM	4 mL
SDS (20% w/v)	0.75%	3.75 mL
ddH_2_O	N/A	77.25 mL
**Total**	**N/A**	**100 mL**

Store at room temperature (25°C) for 12 months.

• DNA elution buffer: Add 20 μL Proteinase K to 200 μL DNA elution stock buffer. Prepare fresh on the day and keep at room temperature (25°C).
• 1x CutSmart buffer: dilute the 10x CutSmart buffer (New England Biolabs) in ddH_2_O. Prepare fresh on the day and keep on ice.

***Note:*** New England Biolabs has recently changed their CutSmart buffer to use recombinant BSA, called rCutSmart. Both CutSmart and rCutSmart should work for this protocol.

## Step-by-step method details

### Overexpression of fission yeast condensin

**Timing: 2.5 days**

This part of the protocol details the growth conditions to overexpress condensin and cell harvest.

1. Wake up condensin overexpression strain from 70°C glycerol stock.
  a. Streak the condensin-expressing budding yeast strain from the −70°C glycerol stock onto a YPD (Yeast Peptone medium supplemented with 2% glucose) agar plate.
  b. Incubate the plate at 25°C overnight (16 h).

***Note:*** We recommend growing up budding yeast freshly from the glycerol stock for large-scale culture and subsequent protein purification.

2. On the next day, inoculate 50 mL YPD medium with the condensin-expressing strain and shake at 25°C for 4–8 h.

***Note:*** Do not allow this pre-culture to grow beyond OD_600_ = 1.5.

3. Inoculate 2 L YPR medium with the required pre-culture volume to reach an OD_600_ of 0.6 on the next morning.

***Note:*** We recommend inoculating the 2 L YPR medium on the night before and culturing the cells at 25°C overnight (between 12 -14 h) until the next morning. This will allow sufficient time for the yeast to adapt to the raffinose as the carbon source.

***Note:*** The doubling time of the condensin-expressing budding yeast is 2 h in our laboratory but may differ depending on local variation of media and growth conditions. Use the measured doubling time to calculate the amount of pre-culture to inoculate the 2 L cultures. We recommend inoculating four 2 L YPR cultures for better yield.

4. On the next day, when the OD_600_ of the cultures reaches around 0.6, induce condensin overexpression by addition of 0.2 L filtered 20% galactose per 2 L culture.
5. Continue shaking the culture at 25°C for 4 h.
6. Collect cells by centrifugation in a JLA8.1 rotor at 3500 rpm (or 3500 × *g*) at 4°C for 10 min.

***Note:*** The centrifuge bottles can be reused to collect cell cultures.

7. Resuspend all of the cell pellet in 0.6 L ice-cold ddH_2_O.
8. Centrifuge again in a JLA8.1 rotor at 3500 × *g* at 4°C for 10 min.
9. Resuspend the cells in 50 mL IgG lysis buffer.
10. Using a 10 mL pipette tip, drip the cell suspension drop by drop into liquid nitrogen.
11. Collect the flash frozen cell suspensions in a beaker and store at −70°C.

**Pause point:** The flash-frozen yeast cells can be stored at −70°C for a few months.

### Purification of fission yeast condensin

**Timing: 2 days**

This part of the protocol details the condensin purification from the harvested yeast cells.

12. Break the cells by grinding under liquid nitrogen, e.g., using a Cole-Parmer cryogenic Freezer/Mill using its standard protocol for yeast lysis (6 cycles of 1 min cooling and 2 min of cell grinding at 15 cps).

***Note:*** The tubes for the Freezer/Mill should not be more than two thirds filled.

13. Thaw the yeast powder at 4°C and mix well with 100 mL IgG lysis buffer.

***Note:*** Ensure cells are completely thawed and no clumps remains.

14. Clear the lysate by centrifugation at 100,000 × *g* (e.g., 35,000 rpm in a Beckman 45Ti rotor) at 4°C for 45 min.
15. Carefully transfer the cleared lysate to a clean 200 mL Duran bottle.

***Note:*** Avoid the white flurry layer at the top and the brown viscous layer at the bottom.

16. Add 2 mL Rabbit IgG-Agarose beads (4 mL beads suspension) to the cleared lysate derived from 8 L culture.
17. Seal the bottle with its cap and roll at 4°C for 1 h.
18. Pour the incubated beads into a clean gravity flow column, e.g., Econo-Pac column from Bio-Rad.
19. Wash the beads once with 20 mL IgG wash buffer.

***Optional:*** After the first wash, we recommend washing the beads once with 10 mL IgG wash buffer supplemented with 10 mM MgCl_2_ and 1 mM ATP. This wash helps remove chaperone proteins that might associate with the overexpressed condensin.

20. Wash the beads six times with 20 mL IgG wash buffer.
21. Resuspend beads in 20 mL IgG wash buffer and transfer to a clean 50 mL tube.
22. Add to the suspension 40 μg 3C protease to cleave off the Protein A tag for protein elution and 40 μg RNase A to facilitate protein elution. Seal the tube and roll at 4°C overnight (16 h).
23. Next day, pour the beads suspension onto a clean gravity column. Collect the flow through which contains the eluted condensin (Figure 2A). Troubleshooting 1.

**Figure 2:**
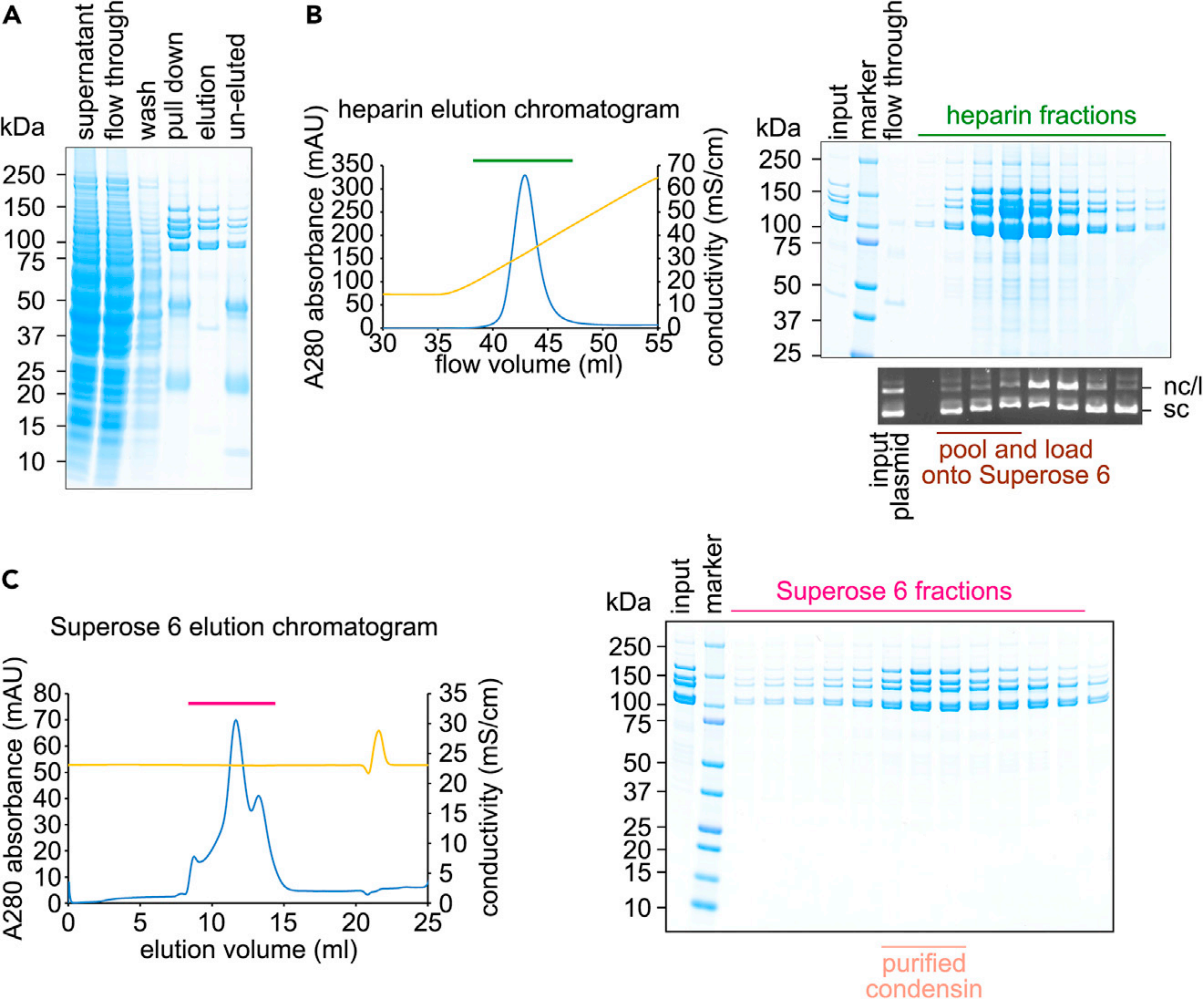
Purification of the fission yeast condensin complex (A) SDS-PAGE analysis followed by Coomassie Blue staining of the protein samples taken from the indicated steps of the IgG affinity pull down. (B) Heparin purification step. Left: chromatogram of the heparin purification step. Blue line, absorbance at 280 nm. Yellow line, conductivity. Green line, fractions collected for SDS-PAGE and nuclease activity analyses. Right: SDS-PAGE analysis of the indicated fractions from the heparin elution (top), and the results from a nuclease activity assay of the corresponding fractions (bottom). nc, nicked circular; l, linear; sc, supercoiled circular. Brown line, fractions collected for concentration and further purification. (C) Superose 6 size exclusion step. Left: chromatogram of the Superose 6 purification step. Blue line, absorbance at 280 nm. Yellow line, conductivity. Pink line, fractions collected for SDS-PAGE analysis. Right: SDS-PAGE analysis of the indicated fractions from the Superose 6 elution. Light pink line, the pooled fractions of purified condensin.
24. Using a liquid chromatography system, e.g., AKTA system, load the supernatant onto a 1 mL Hi-Trap Heparin column, pre-equilibrated with Heparin A buffer.
25. Wash the Heparin column with 20 mL Heparin A buffer.
26. Elute condensin with a 20 mL linear gradient from Heparin A to Heparin B buffer. Collect 0.5 mL fractions.

***Optional:*** In addition to taking samples from each fraction for SDS-PAGE analysis, we recommend testing each fraction for nuclease contamination. We incubate 10 μL from each fraction with 50 ng supercoiled plasmid, such as pBlueScript, in the presence of 10 mM MgCl_2_ at 30°C for 1 h. Then each sample is supplemented with 0.5% SDS and 1 μg/mL Proteinase K followed by incubation at 50°C for 30 min. Finally, we separate DNA on a 0.8% agarose gel and stain with GelRed. Loss of the plasmid, or appearance of nicked or linear species, is indicative of contaminating nuclease activity.

27. Pool the peak fractions that are free of nuclease (Figure 2B). Troubleshooting 2.
28. Concentrate the condensin sample by ultrafiltration e.g., in a Vivaspin 6 concentrator, to 0.5 mL Troubleshooting 1.
29. Load the concentrated condensin sample onto a Superose 6 gel filtration column pre-equilibrated with Gel filtration buffer. Collect 0.25 mL fractions.
30. Store the purified condensin.
  a. Pool the peak fractions (Figure 2C).
  b. Concentrate condensin, again by ultrafiltration, to around 1 mg/mL.
  c. Prepare aliquots and flash freeze those in liquid nitrogen.
  d. Store at −70°C until use.

**Pause point:** The flash-frozen purified condensin can be stored at −70°C for a few months.

### Preparation of the digoxygenin-labeled linear dsDNA substrate

**Timing: 6 h**

This step of the protocol details the preparation of digoxygenin-labeled dsDNA substrate (dig-DNA).

31. Preparation of the DNA substrate is based on triple digoxygenin-labeled oligonucleotide DNA primers (MT392 and MT393), ordered from a primer synthesis company, e.g., Integrated DNA Technologies.

**CRITICAL:** Each of the two primers should be triply digoxygenin-labeled for reliable binding to the beads. Singly digoxygenin-labeled primers were found to detach during incubations.

32. PCR amplify the template plasmid (pEGFP-C1, ClonTech) with oligonucleotide primers MT462 and MT463, e.g., using CloneAmp PCR reagents according to manufacturer’s instructions.
33. After separating the amplified DNA on 0.8% Tris-Acetate-EDTA agarose gel, gel purify the amplified DNA, e.g., using Nucleospin gel and PCR cleanup according to manufacturer’s instructions.
34. PCR amplify the purified DNA product from the first round of amplification using digoxygenin-labeled oligos MT392 and MT393, e.g., using CloneAmp PCR reagents according to manufacturer’s instructions..
35. After separating the amplified DNA on 0.8% Tris-Acetate-EDTA agarose gel, gel purify the amplified DNA, e.g., using Nucleospin Gel and PCR cleanup according to manufacturer’s instructions.
36. Measure the yield and concentration using Nanodrop spectrophotometry.

**Pause point:** The purified dig-DNA (digoxygenin-labelled dsDNA) can be stored at −20°C for a year.

### Condensin loading and second dsDNA capture

**Timing: 4 h**

***Note:*** This section of the protocol must be immediately followed by the protocols detailed in the next sections, which takes an additional 4–6 h.

**Figure 3:**
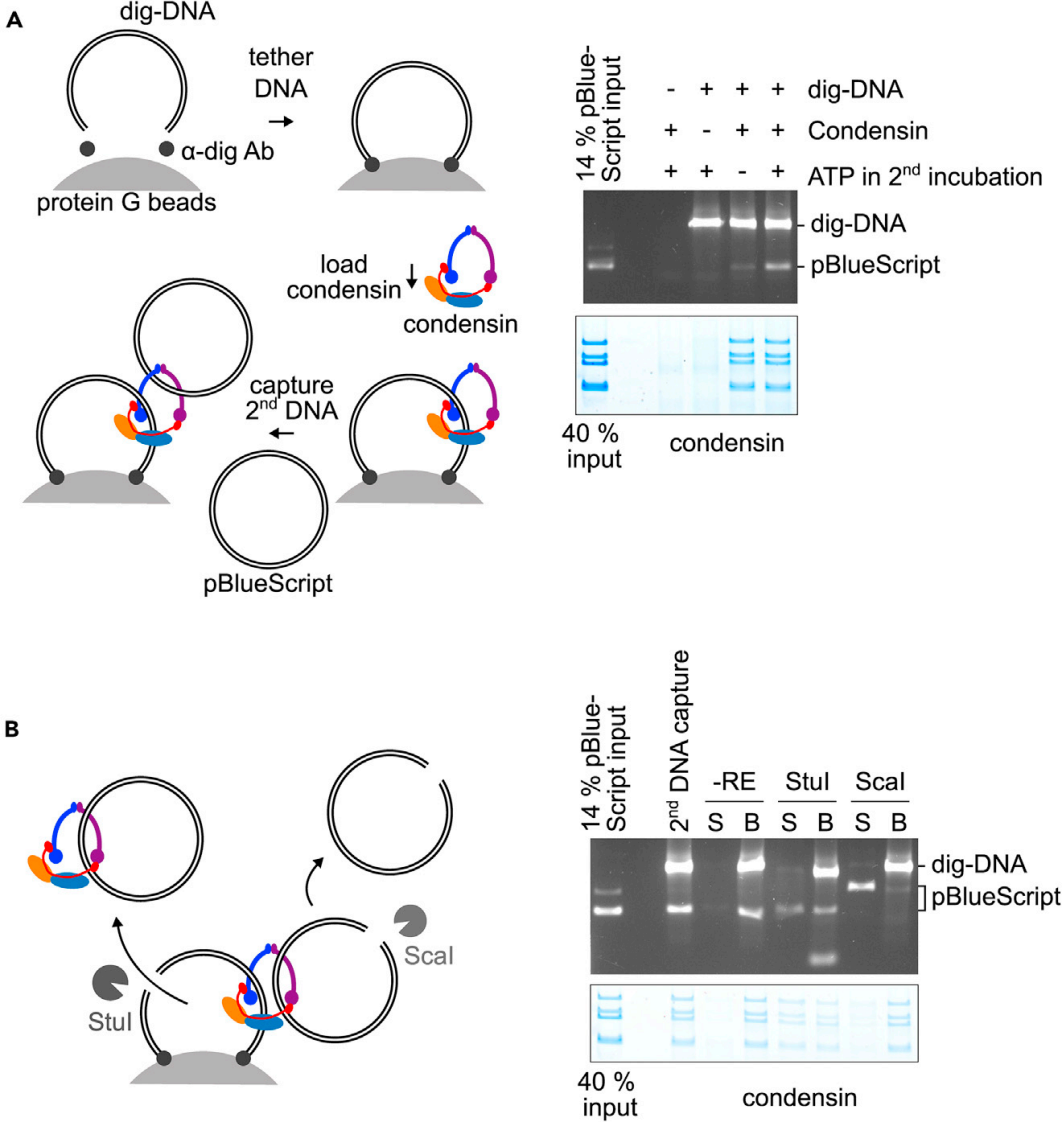
Second DNA capture reaction using purified condensin (A) Second DNA capture followed by direct sample analysis. Left: schematic of the workflow for a second DNA capture reaction. The first DNA substrate is assembled by tethering digoxygenin-labeled dsDNA (dig-DNA) onto protein G magnetic beads via an α-digoxygenin antibody (α-dig Ab). The first DNA substrate is then incubated with condensin for topological loading. After washing off the excess condensin complex, the beads are further incubated with the second pBlueScript dsDNA plasmid. Right: DNA agarose gel analysis (top) and protein SDS-PAGE analysis (bottom) of the bead-bound fraction after the second DNA capture reaction are shown. (B) Restriction enzyme cleavage to probe the topological nature of the condensin-DNA interaction. Left: a schematic illustrates the expected outcomes of restriction enzyme cleavage using StuI or ScaI, respectively. Right: DNA agarose gel analysis (top) and protein SDS-PAGE analysis (bottom) of both the supernatant (S) and bead-bound (B) fractions after treatment of a 2^nd^ DNA capture reaction without restriction enzyme (-RE), or the indicated enzymes.

This step of the protocol details the condensin loading and second dsDNA capture reactions. We first couple the dig-DNA via α-digoxygenin antibodies to magnetic beads that can be easily washed and collected using the DynaMag magnet. We then perform the condensin loading reaction. After washing off excess condensin, we incubate the beads with pBlueScript plasmid to initiate the second dsDNA capture reaction. Finally, we use buffer with high ionic strength to remove condensin or pBlueScript plasmids that are not stably bound to the digoxygenin-labeled dsDNA on the beads (Figure 3A).

***Note:*** Unless stated otherwise, all buffers in this step should be kept on ice throughout the experiment.

37. For x number of loading reactions, pipette 10x μL protein G coupled Dynabeads suspension into a clean low-binding microcentrifuge tube.
38. Wash the protein A coupled Dynabeads in 1 mL PBSA-BSA buffer twice.

***Note:*** All subsequent steps regarding washing the protein A coupled Dynabeads refers to the following procedure. Resuspend beads in the indicated amount of buffer by carefully inverting the tubes multiple times until no clumps remain. Then immediately use a magnet to collect the Dynabeads at the side wall of the tube. Aspirate or pipette off the buffer.

39. Resuspend the beads in 100x μL PBSA-BSA buffer and constantly rotate the tube at 4°C for 20 min.
40. Mix 2x μL DNA binding buffer with 0.3x μL anti-digoxygenin antibody and 100x ng digoxygenin-labeled linear dsDNA. Incubate the reaction at room temperature (25°C) for 30 min.
41. After incubation, add the antibody/DNA mix to the Dynabeads suspension and continue rotating the tube at 4°C for at least 1.5 h.
42. Wash the beads once with 1 mL PBSA-BSA buffer, twice with 1 mL Loading wash buffer, and once with 1 mL Loading pre-equilibration buffer.
43. Resuspend beads in 100x μL Loading pre-equilibration buffer and aliquot suspension into x low-binding microcentrifuge tubes.
44. Collect beads using a magnet. Remove supernatant as much as possible.
45. Resuspend beads in 15 μL Loading reaction buffer supplemented with 200 nM purified condensin and 1 mM ATP.
46. Incubate and shake the tubes with 850 rpm at 30°C for 30 min in a thermomixer. Completely resuspend the beads by tapping the tubes every 5 min to avoid beads aggregating at the bottom of the tubes. Troubleshooting 3.

***Optional:*** After the incubation, if you are interested in the unbound fraction, collect beads using a magnet and take sample from the supernatant for DNA and/or protein analyses as detailed the section below.

47. Wash the beads three times with 1 mL Loading pre-equilibration buffer.
48. Resuspend beads in 0.1 mL Loading pre-equilibration buffer.
49. Collect beads using a magnet and discard the supernatant.
50. Resuspend beads in 15 μL Loading reaction buffer supplemented with 1 mM ATP and 100 ng pBlueScript supercoiled dsDNA plasmid.
51. Incubate and shake the tubes with 850 rpm at 30°C for 30 min in a thermomixer. Completely resuspend the beads by tapping the tubes every 5 min to avoid beads aggregating at the bottom of the tubes.

***Optional:*** After the incubation, if you are interested in the unbound fraction, collect beads using a magnet and take sample from the supernatant for DNA and/or protein analyses as detailed the section below.

52. Wash the beads three times with 1 mL Loading wash buffer and once with 1 mL Loading pre-equilibration buffer.

### Sample analyses after the second dsDNA capture reaction

**Timing: 4 h**

After the condensin second dsDNA capture reaction, immediately follow the protocol in this section to analyze the bead-bound protein and DNA components.

53. Resuspend beads in 1 mL Loading pre-equilibration buffer.
  a. Take 0.7 mL sample from the suspension for DNA analysis.
  b. Take 0.25 mL sample from the suspension for protein analysis.
  c. For both samples, collect beads using a magnet and remove the supernatant.
54. To analyze protein content.
  a. To each sample, add 10 μL SDS sample buffer.
  b. Boil samples at 95°C for 5 min.
  c. Collect beads using a magnet and apply the supernatant for Tris-Glycine protein gel electrophoresis followed by Coomassie Blue staining. Troubleshooting 4.
55. To analyze DNA content.
  a. To each sample, add 10 μL Loading DNA elution buffer.
  b. Incubate samples at 50°C for at least 20 min with vigorous shaking.
  c. Collect beads using a magnet and load the supernatant for TAE-agarose gel electrophoresis followed by GelRed staining. Troubleshooting 5.

### Restriction digestion of the reaction products

**Timing: 2 h**

To investigate topology of the product after condensin second dsDNA capture reaction, we use restriction enzymes to specifically cut either the digoxygenin-labeled linear dsDNA or the supercoiled pBlueScript dsDNA plasmid, then analyze the DNA and protein contents on the beads and in the supernatant (Figure 3B).

56. Resuspend beads in 0.1 mL Loading pre-equilibration buffer.
57. Collect beads using a magnet and resuspend beads in 10 μL 1x CutSmart buffer with or without 1 μL StuI or 1 μL ScaI-HF.
58. Incubate the tubes at 18°C and shake at 1100 rpm in a thermomixer for 1 h.
59. Cool the reaction on ice and add 10 μL 1x CutSmart buffer supplemented with 1 M NaCl. Resuspend well by tapping the tubes.
60. Take 14 μL for DNA analyses and 5 μL for protein analyses. Collect the beads using a magnet and keep the supernatant for downstream analyses.
61. Analyze the DNA and protein samples according to steps detailed in the section above.

## Expected outcomes

Before we begin, we construct a fission yeast condensin over-expression strain by integrating three plasmids (Figure 1A) sequentially into the budding yeast host. Since the protein A tag, which is fused to Cnd2 from the second plasmid, binds to any antibody, the integration of the first two plasmids can be confirmed simultaneously on Western blot of the whole cell extract after galactose induction of protein expression using an α-Pk antibody (Figure 1B). Integration and galactose-induced protein expression from the third plasmid must be confirmed using a small-scale IgG pull down. This step is crucial because of the various expression or subunit incorporation levels of Cnd1 among different clones (Figure 1C).

The final expression strain is then cultured in a large volume before galactose induction. Fission yeast condensin is then purified using a three-step protocol, consisting of IgG pull down, heparin affinity chromatography, and a Superose 6 size exclusion chromatography step. Small samples of protein can be collected from each step and analyzed by SDS-PAGE (Figure 2). From a typical 8 L cell culture harvested with a final OD_600_ between 1.5 and 2, we expect to obtain between 200 μg to 300 μg purified fission yeast condensin complex at a concentration of over 1 mg/mL.

Using the purified condensin, we perform a DNA loading and second DNA capture reaction. We split the beads in our reactions to analyze both the DNA and protein content remaining on the beads after high-salt buffer washes. The expected recovery of condensin on the bead-bound DNA should be between 20%‒40% of the input. The expected recovery of the second DNA is between 5%-10% of the input. Note that condensin will also undergo enzymatic unloading from DNA in the presence of ATP, especially when incubated at elevated ionic strengths.^1^ The low ionic strength Loading reaction buffer described in this protocol is designed to favor loading over unloading (Figure 3A). The expected results from restriction enzyme treatment of the second DNA capture products are as follows. In an incubation without restriction enzyme, both dig-DNA and pBlueScript second DNA remain in the bead-bound fraction. StuI treatment should release a proportional amount of condensin and pBlueScript DNA into the supernatant, while the two dig-DNA fragments after StuI treatment should remain bead-bound. ScaI treatment in turn should release the linearized pBlueScript DNA into the supernatant, while the intact dig-DNA and condensin should remain in the bead-bound fraction (Figure 3B).

## Limitations

This protocol uses high-salt resistance of the condensin-DNA interaction as an indicator of its topological nature, together with the requirement of a topologically closed DNA substrate. Such a relatively simple setup has advantages, such as minimal protein perturbation, and easy handling. However, this approach does not address where inside the condensin complex the DNA is entrapped. It also cannot distinguish between topological and pseudo-topological entrapment (where the DNA traverses the condensin ring once or twice, respectively). Additional experiments, such as protein interface crosslinking,^6^ are needed to investigate these possibilities.

Another limitation of the described bulk biochemical approach is that it cannot distinguish whether a single condensin complex, or multiple condensins together, hold together two DNA substrates. Such information can be obtained when adapting the above-described reagents and reactions to a single molecule microscopy format. The latter method requires fluorescently labeled condensin complexes for visualization.^1^

The described protocol is suitable not only for investigating condensin-DNA interactions, but also those of other SMC family members such as cohesin, the SMC5-SMC6 complex, bacterial or archaeal SMC complexes, as well as more distant relatives like the Rad50 complex. While the required adaptations should be straight forward, the stability of the respective protein complexes to high-salt buffer treatment will need to be explored. The stringency of the washing steps should be adjusted empirically to avoid inadvertently stripping proteins from the bead-bound DNA.

## Troubleshooting

### Problem 1

Low yield from condensin purification.

We expect to obtain about 200–300 μg purified fission yeast condensin complex from 8 L yeast cell culture. However, improper handling during the purification steps could reduce the final yield.

### Potential solution

• A good purification starts with the cell culture. We recommend always keeping the OD_600_ of the cell culture below 1.5, both in the pre-culture and the large YPR culture (Step 2 and Step 4) before induction of protein expression.
• Condensin shows a tendency to aggregate when exposed to sudden ionic strength changes. Load eluates from the IgG beads directly onto the Heparin column (Step 24). Concentrate the heparin eluate and load directly onto the Superose 6 column (Step 29). Do not adjusting the salt concentration in either case.
• After pooling condensin-containing fractions after Heparin chromatography (Step 28), we expect 5–6 mL of sample at 0.1–0.5 mg/mL concentration, which needs to be concentrated before size exclusion chromatography. Reducing the volume to 0.5 mL, the recommended sample volume for a 24 mL Superose 6 column (Cytiva), might result in condensin aggregation and a reduction of the final yield. We recommend concentrating the sample to 1 mL before loading onto the Superose 6 column, which resulted in no noticeable loss of resolution during size exclusion chromatography.

### Problem 2

Nuclease contamination in the condensin preparation (related to Step 27). Unidentified nuclease activity might contaminate the purified condensin, leading to unexpected and unexplainable results in the condensin loading reactions.

### Potential solution

We traced nuclease activity throughout the condensin purification and found that it is best separated in the salt gradient elution step during heparin chromatography. The nuclease activity peak is separated by at least one fraction from the condensin peak (Figure 2B). Therefore, we recommend eluting the condensin from the heparin affinity column using a linear salt gradient, and assay each fraction for nuclease contamination before deciding which fractions to pool for downstream size exclusion chromatography (related to the optional step between Steps 26 and 27).

### Problem 3

Inconsistent condensin recovery, as observed by SDS-PAGE analysis, after a second DNA capture reaction. This problem might occur when condensin is loaded onto bead-bound dig-DNA and the resultant beads are then split to perform the second DNA capture reaction.

### Potential solution

This problem usually results from bead aggregation inside the test tube. Clumping becomes apparent especially during the washing steps.

• If not kept thoroughly resuspended, beads might aggregate after incubation with condensin (related to Step 46). We recommend frequently checking the test tubes during the condensin loading step and to manually resuspend the beads should bead sedimentation become apparent.
• Repeated freeze thaw cycles can aggregate purified condensin, exacerbating bead aggregation and reducing biochemical activity. We recommend using fresh condensin aliquots thawed only once from −70°C storage for best results.

### Problem 4

Inefficient condensin recovery, as observed by SDS-PAGE analysis after a second DNA capture reaction (related to Step 54).

### Potential solution

• Condensin that experienced many freeze thaw cycles, or stored for more than 6 months, might present reduced loading efficiency, as well as inefficient second DNA capture. Always use fresh condensin for second DNA capture experiments (related to Step 46).
• Digoxygenin-labeled DNA (dig-DNA) might not be stably double-tethered to the beads, if either end contains less than three digoxygenin labels. In this case, topologically loaded condensin will slide off beads during the high-salt wash step. Always use triply digoxygenin-labeled primers to produce dig-DNA (related to Step 31).
• Dig-DNA might be singly tethered to the beads if either the α-digoxygenin antibody or the protein G magnetic beads are compromised, resulting in condensin sliding off the dig-DNA during high-salt washes. Always use reagent concentrations as specified in the protocol (related to Steps 37 and 40).

### Problem 5

Little or no second dsDNA captured by condensin (related to Step 55).

### Potential solution

• Condensin that experienced many freeze thaw cycles, or stored for more than 6 months, might present reduced loading efficiency, as well as inefficient second DNA capture. Always use fresh condensin for second DNA capture experiments (related to Step 46).
• Remnant salt from washing steps might compromise the second DNA loading reaction. Since condensin loading and second DNA capture is salt-sensitive, we recommend completely removing the Loading pre-equilibration buffer after the intermediate washing step (related to Steps 48 and 49).
• Insufficient condensin loading onto the dig-DNA will reduce second DNA capture. We recommend using 200 nM of condensin during the first loading step to obtain efficient second DNA capture results (related to Step 46).

## Resource availability

### Lead contact

Further information and requests for resources and reagents should be directed to and will be fulfilled by the lead contact, Frank Uhlmann (frank.uhlmann@crick.ac.uk).

### Technical contact

Questions about the technical specifics of performing the protocol should be directed to and will be answered by the technical contact, Minzhe Tang (minzhe.tang@crick.ac.uk).

### Materials availability

Plasmids, recombinant proteins, DNA substrates are available without restriction upon requests, which should be directed to the lead contact, Frank Uhlmann (frank.uhlmann@crick.ac.uk).

### Data and code availability

This study did not generate or analyze any datasets.
